# Supplementary material for: A fault-intrusion-tolerant system and deadline-aware algorithm for scheduling scientific workflow in the cloud
Source: PeerJ Comput Sci. 2021 Nov 2;7:e747. doi: 10.7717/peerj-cs.747 (PMC8576554; doi:10.7717/peerj-cs.747)
Supplement: Supplemental Information 6 [file peerj-cs-07-747-s006.zip › Myproject1.1/docs/org/cloudbus/cloudsim/network/datacenter/NetworkCloudletSpaceSharedScheduler.html]

NetworkCloudletSpaceSharedScheduler


JavaScript is disabled on your browser.


- Overview
- Package
- Class
- Use
- Tree
- Deprecated
- Index
- Help

- Prev Class
- Next Class

- Frames
- No Frames

- All Classes

- Summary:
- Nested |
- Field |
- Constr |
- Method

- Detail:
- Field |
- Constr |
- Method


org.cloudbus.cloudsim.network.datacenter

## Class NetworkCloudletSpaceSharedScheduler

- java.lang.Object
- - org.cloudbus.cloudsim.CloudletScheduler
  - - org.cloudbus.cloudsim.network.datacenter.NetworkCloudletSpaceSharedScheduler

- ---

    

  ```
  public class NetworkCloudletSpaceSharedScheduler
  extends CloudletScheduler
  ```

  CloudletSchedulerSpaceShared implements a policy of scheduling performed by a virtual machine. It
  consider that there will be only one cloudlet per VM. Other cloudlets will be in a waiting list.
  We consider that file transfer from cloudlets waiting happens before cloudlet execution. I.e.,
  even though cloudlets must wait for CPU, data transfer happens as soon as cloudlets are
  submitted.

  Since:
  :   CloudSim Toolkit 3.0

- - ### Field Summary

    Fields

    | Modifier and Type | Field and Description |
    | --- | --- |
    | `protected int` | `currentCpus` The current CPUs. |
    | `java.util.Map<java.lang.Integer,java.util.List<HostPacket>>` | `pktrecv` |
    | `java.util.Map<java.lang.Integer,java.util.List<HostPacket>>` | `pkttosend` |
    | `protected int` | `usedPes` The used PEs. |
  - ### Constructor Summary

    Constructors

    Constructor and Description

    `NetworkCloudletSpaceSharedScheduler()`

    Creates a new CloudletSchedulerSpaceShared object.
  - ### Method Summary

    Methods

    | Modifier and Type | Method and Description |
    | --- | --- |
    | `Cloudlet` | `cloudletCancel(int cloudletId)` Cancels execution of a cloudlet. |
    | `void` | `cloudletFinish(ResCloudlet rcl)` Processes a finished cloudlet. |
    | `boolean` | `cloudletPause(int cloudletId)` Pauses execution of a cloudlet. |
    | `double` | `cloudletResume(int cloudletId)` Resumes execution of a paused cloudlet. |
    | `double` | `cloudletSubmit(Cloudlet cloudlet)` Receives an cloudlet to be executed in the VM managed by this scheduler. |
    | `double` | `cloudletSubmit(Cloudlet cloudlet, double fileTransferTime)` Receives an cloudlet to be executed in the VM managed by this scheduler. |
    | `protected <T extends ResCloudlet>  void` | `cloudletWaitingList(java.util.List<T> cloudletWaitingList)` Cloudlet waiting list. |
    | `protected <T extends ResCloudlet>  java.util.List<T>` | `getCloudletExecList()` Gets the cloudlet exec list. |
    | `protected <T extends ResCloudlet>  java.util.List<T>` | `getCloudletFinishedList()` Gets the cloudlet finished list. |
    | `protected <T extends ResCloudlet>  java.util.List<T>` | `getCloudletPausedList()` Gets the cloudlet paused list. |
    | `int` | `getCloudletStatus(int cloudletId)` Gets the status of a cloudlet. |
    | `protected <T extends ResCloudlet>  java.util.List<T>` | `getCloudletWaitingList()` Gets the cloudlet waiting list. |
    | `java.util.List<java.lang.Double>` | `getCurrentRequestedMips()` Gets the current requested mips. |
    | `double` | `getCurrentRequestedUtilizationOfBw()` Gets the current requested bw. |
    | `double` | `getCurrentRequestedUtilizationOfRam()` Gets the current requested ram. |
    | `Cloudlet` | `getNextFinishedCloudlet()` Returns the next cloudlet in the finished list, $null if this list is empty. |
    | `double` | `getTotalCurrentAllocatedMipsForCloudlet(ResCloudlet rcl, double time)` Gets the total current allocated mips for cloudlet. |
    | `double` | `getTotalCurrentAvailableMipsForCloudlet(ResCloudlet rcl, java.util.List<java.lang.Double> mipsShare)` Gets the total current mips for the Cloudlet. |
    | `double` | `getTotalCurrentRequestedMipsForCloudlet(ResCloudlet rcl, double time)` Gets the total current requested mips for cloudlet. |
    | `double` | `getTotalUtilizationOfCpu(double time)` Get utilization created by all cloudlets. |
    | `boolean` | `isFinishedCloudlets()` Informs about completion of some cloudlet in the VM managed by this scheduler. |
    | `Cloudlet` | `migrateCloudlet()` Returns one cloudlet to migrate to another vm. |
    | `int` | `runningCloudlets()` Returns the number of cloudlets runnning in the virtual machine. |
    | `protected <T extends ResCloudlet>  void` | `setCloudletExecList(java.util.List<T> cloudletExecList)` Sets the cloudlet exec list. |
    | `protected <T extends ResCloudlet>  void` | `setCloudletFinishedList(java.util.List<T> cloudletFinishedList)` Sets the cloudlet finished list. |
    | `protected <T extends ResCloudlet>  void` | `setCloudletPausedList(java.util.List<T> cloudletPausedList)` Sets the cloudlet paused list. |
    | `double` | `updateVmProcessing(double currentTime, java.util.List<java.lang.Double> mipsShare)` Updates the processing of cloudlets running under management of this scheduler. |

    - ### Methods inherited from class org.cloudbus.cloudsim.CloudletScheduler

      `getCurrentMipsShare, getPreviousTime, setCurrentMipsShare, setPreviousTime`
    - ### Methods inherited from class java.lang.Object

      `clone, equals, finalize, getClass, hashCode, notify, notifyAll, toString, wait, wait, wait`

- - ### Field Detail


    - #### currentCpus

      ```
      protected int currentCpus
      ```

      The current CPUs.


    - #### usedPes

      ```
      protected int usedPes
      ```

      The used PEs.


    - #### pkttosend

      ```
      public java.util.Map<java.lang.Integer,java.util.List<HostPacket>> pkttosend
      ```


    - #### pktrecv

      ```
      public java.util.Map<java.lang.Integer,java.util.List<HostPacket>> pktrecv
      ```
  - ### Constructor Detail


    - #### NetworkCloudletSpaceSharedScheduler

      ```
      public NetworkCloudletSpaceSharedScheduler()
      ```

      Creates a new CloudletSchedulerSpaceShared object. This method must be invoked before
      starting the actual simulation.
  - ### Method Detail


    - #### updateVmProcessing

      ```
      public double updateVmProcessing(double currentTime,
                              java.util.List<java.lang.Double> mipsShare)
      ```

      Updates the processing of cloudlets running under management of this scheduler.

      **Specified by:**
      :   `updateVmProcessing` in class `CloudletScheduler`

      Parameters:
      :   `currentTime` - current simulation time
      :   `mipsShare` - array with MIPS share of each processor available to the scheduler

      Returns:
      :   time predicted completion time of the earliest finishing cloudlet, or 0 if there is
          no next events


    - #### cloudletCancel

      ```
      public Cloudlet cloudletCancel(int cloudletId)
      ```

      Cancels execution of a cloudlet.

      **Specified by:**
      :   `cloudletCancel` in class `CloudletScheduler`

      Parameters:
      :   `cloudletId` - ID of the cloudlet being cancealed

      Returns:
      :   the canceled cloudlet, $null if not found


    - #### cloudletPause

      ```
      public boolean cloudletPause(int cloudletId)
      ```

      Pauses execution of a cloudlet.

      **Specified by:**
      :   `cloudletPause` in class `CloudletScheduler`

      Parameters:
      :   `cloudletId` - ID of the cloudlet being paused

      Returns:
      :   $true if cloudlet paused, $false otherwise


    - #### cloudletFinish

      ```
      public void cloudletFinish(ResCloudlet rcl)
      ```

      Processes a finished cloudlet.

      **Specified by:**
      :   `cloudletFinish` in class `CloudletScheduler`

      Parameters:
      :   `rcl` - finished cloudlet


    - #### cloudletResume

      ```
      public double cloudletResume(int cloudletId)
      ```

      Resumes execution of a paused cloudlet.

      **Specified by:**
      :   `cloudletResume` in class `CloudletScheduler`

      Parameters:
      :   `cloudletId` - ID of the cloudlet being resumed

      Returns:
      :   $true if the cloudlet was resumed, $false otherwise


    - #### cloudletSubmit

      ```
      public double cloudletSubmit(Cloudlet cloudlet,
                          double fileTransferTime)
      ```

      Receives an cloudlet to be executed in the VM managed by this scheduler.

      **Specified by:**
      :   `cloudletSubmit` in class `CloudletScheduler`

      Parameters:
      :   `cloudlet` - the submited cloudlet
      :   `fileTransferTime` - time required to move the required files from the SAN to the VM

      Returns:
      :   expected finish time of this cloudlet, or 0 if it is in the waiting queue


    - #### cloudletSubmit

      ```
      public double cloudletSubmit(Cloudlet cloudlet)
      ```

      **Description copied from class: `CloudletScheduler`**

      Receives an cloudlet to be executed in the VM managed by this scheduler.

      **Specified by:**
      :   `cloudletSubmit` in class `CloudletScheduler`

      Parameters:
      :   `cloudlet` - the submited cloudlet

      Returns:
      :   expected finish time of this cloudlet, or 0 if it is in a waiting queue


    - #### getCloudletStatus

      ```
      public int getCloudletStatus(int cloudletId)
      ```

      Gets the status of a cloudlet.

      **Specified by:**
      :   `getCloudletStatus` in class `CloudletScheduler`

      Parameters:
      :   `cloudletId` - ID of the cloudlet

      Returns:
      :   status of the cloudlet, -1 if cloudlet not found


    - #### getTotalUtilizationOfCpu

      ```
      public double getTotalUtilizationOfCpu(double time)
      ```

      Get utilization created by all cloudlets.

      **Specified by:**
      :   `getTotalUtilizationOfCpu` in class `CloudletScheduler`

      Parameters:
      :   `time` - the time

      Returns:
      :   total utilization


    - #### isFinishedCloudlets

      ```
      public boolean isFinishedCloudlets()
      ```

      Informs about completion of some cloudlet in the VM managed by this scheduler.

      **Specified by:**
      :   `isFinishedCloudlets` in class `CloudletScheduler`

      Returns:
      :   $true if there is at least one finished cloudlet; $false otherwise


    - #### getNextFinishedCloudlet

      ```
      public Cloudlet getNextFinishedCloudlet()
      ```

      Returns the next cloudlet in the finished list, $null if this list is empty.

      **Specified by:**
      :   `getNextFinishedCloudlet` in class `CloudletScheduler`

      Returns:
      :   a finished cloudlet


    - #### runningCloudlets

      ```
      public int runningCloudlets()
      ```

      Returns the number of cloudlets runnning in the virtual machine.

      **Specified by:**
      :   `runningCloudlets` in class `CloudletScheduler`

      Returns:
      :   number of cloudlets runnning


    - #### migrateCloudlet

      ```
      public Cloudlet migrateCloudlet()
      ```

      Returns one cloudlet to migrate to another vm.

      **Specified by:**
      :   `migrateCloudlet` in class `CloudletScheduler`

      Returns:
      :   one running cloudlet


    - #### getCloudletWaitingList

      ```
      protected <T extends ResCloudlet> java.util.List<T> getCloudletWaitingList()
      ```

      Gets the cloudlet waiting list.

      Type Parameters:
      :   `T` - the generic type

      Returns:
      :   the cloudlet waiting list


    - #### cloudletWaitingList

      ```
      protected <T extends ResCloudlet> void cloudletWaitingList(java.util.List<T> cloudletWaitingList)
      ```

      Cloudlet waiting list.

      Type Parameters:
      :   `T` - the generic type

      Parameters:
      :   `cloudletWaitingList` - the cloudlet waiting list


    - #### getCloudletExecList

      ```
      protected <T extends ResCloudlet> java.util.List<T> getCloudletExecList()
      ```

      Gets the cloudlet exec list.

      Type Parameters:
      :   `T` - the generic type

      Returns:
      :   the cloudlet exec list


    - #### setCloudletExecList

      ```
      protected <T extends ResCloudlet> void setCloudletExecList(java.util.List<T> cloudletExecList)
      ```

      Sets the cloudlet exec list.

      Type Parameters:
      :   `T` - the generic type

      Parameters:
      :   `cloudletExecList` - the new cloudlet exec list


    - #### getCloudletPausedList

      ```
      protected <T extends ResCloudlet> java.util.List<T> getCloudletPausedList()
      ```

      Gets the cloudlet paused list.

      Type Parameters:
      :   `T` - the generic type

      Returns:
      :   the cloudlet paused list


    - #### setCloudletPausedList

      ```
      protected <T extends ResCloudlet> void setCloudletPausedList(java.util.List<T> cloudletPausedList)
      ```

      Sets the cloudlet paused list.

      Type Parameters:
      :   `T` - the generic type

      Parameters:
      :   `cloudletPausedList` - the new cloudlet paused list


    - #### getCloudletFinishedList

      ```
      protected <T extends ResCloudlet> java.util.List<T> getCloudletFinishedList()
      ```

      Gets the cloudlet finished list.

      Type Parameters:
      :   `T` - the generic type

      Returns:
      :   the cloudlet finished list


    - #### setCloudletFinishedList

      ```
      protected <T extends ResCloudlet> void setCloudletFinishedList(java.util.List<T> cloudletFinishedList)
      ```

      Sets the cloudlet finished list.

      Type Parameters:
      :   `T` - the generic type

      Parameters:
      :   `cloudletFinishedList` - the new cloudlet finished list


    - #### getCurrentRequestedMips

      ```
      public java.util.List<java.lang.Double> getCurrentRequestedMips()
      ```

      **Description copied from class: `CloudletScheduler`**

      Gets the current requested mips.

      **Specified by:**
      :   `getCurrentRequestedMips` in class `CloudletScheduler`

      Returns:
      :   the current mips


    - #### getTotalCurrentAvailableMipsForCloudlet

      ```
      public double getTotalCurrentAvailableMipsForCloudlet(ResCloudlet rcl,
                                                   java.util.List<java.lang.Double> mipsShare)
      ```

      **Description copied from class: `CloudletScheduler`**

      Gets the total current mips for the Cloudlet.

      **Specified by:**
      :   `getTotalCurrentAvailableMipsForCloudlet` in class `CloudletScheduler`

      Parameters:
      :   `rcl` - the rcl
      :   `mipsShare` - the mips share

      Returns:
      :   the total current mips


    - #### getTotalCurrentAllocatedMipsForCloudlet

      ```
      public double getTotalCurrentAllocatedMipsForCloudlet(ResCloudlet rcl,
                                                   double time)
      ```

      **Description copied from class: `CloudletScheduler`**

      Gets the total current allocated mips for cloudlet.

      **Specified by:**
      :   `getTotalCurrentAllocatedMipsForCloudlet` in class `CloudletScheduler`

      Parameters:
      :   `rcl` - the rcl
      :   `time` - the time

      Returns:
      :   the total current allocated mips for cloudlet


    - #### getTotalCurrentRequestedMipsForCloudlet

      ```
      public double getTotalCurrentRequestedMipsForCloudlet(ResCloudlet rcl,
                                                   double time)
      ```

      **Description copied from class: `CloudletScheduler`**

      Gets the total current requested mips for cloudlet.

      **Specified by:**
      :   `getTotalCurrentRequestedMipsForCloudlet` in class `CloudletScheduler`

      Parameters:
      :   `rcl` - the rcl
      :   `time` - the time

      Returns:
      :   the total current requested mips for cloudlet


    - #### getCurrentRequestedUtilizationOfBw

      ```
      public double getCurrentRequestedUtilizationOfBw()
      ```

      **Description copied from class: `CloudletScheduler`**

      Gets the current requested bw.

      **Specified by:**
      :   `getCurrentRequestedUtilizationOfBw` in class `CloudletScheduler`

      Returns:
      :   the current requested bw


    - #### getCurrentRequestedUtilizationOfRam

      ```
      public double getCurrentRequestedUtilizationOfRam()
      ```

      **Description copied from class: `CloudletScheduler`**

      Gets the current requested ram.

      **Specified by:**
      :   `getCurrentRequestedUtilizationOfRam` in class `CloudletScheduler`

      Returns:
      :   the current requested ram


- Overview
- Package
- Class
- Use
- Tree
- Deprecated
- Index
- Help

- Prev Class
- Next Class

- Frames
- No Frames

- All Classes

- Summary:
- Nested |
- Field |
- Constr |
- Method

- Detail:
- Field |
- Constr |
- Method
